# Prolonged Remission of Cancer of Unknown Primary following Initiation of Eculizumab Therapy for Paroxysmal Nocturnal Hemoglobinuria

**DOI:** 10.1155/2019/2587597

**Published:** 2019-07-02

**Authors:** Ming Y. Lim, Keith E. Volmar, Nigel S. Key

**Affiliations:** ^1^Department of Medicine, Division of Hematology/Oncology, University of North Carolina, Chapel Hill, NC, USA; ^2^Department of Pathology, UNC REX Healthcare, Raleigh, NC, USA

## Abstract

We report the case of a 64-year-old woman who presented with cancer of unknown primary treated with carboplatin and paclitaxel, followed by maintenance erlotinib. Her chemotherapy regimen was discontinued due to the development of profound hemolysis that was later identified to be due to paroxysmal nocturnal hemoglobinuria (PNH). She was started on a complement inhibitory antibody, eculizumab 900 mg every 2 weeks, with marked suppression of hemolysis. Eight years after diagnosis of cancer, the patient remains on eculizumab with no signs of cancer recurrence on regular imaging. Regardless of whether the co-occurrence of cancer and PNH was any more than coincidental in this patient, the uniqueness of the case is emphasized by the remarkable and sustained response of not only PNH but also possibly the associated cancer to eculizumab.

## 1. Introduction

Paroxysmal nocturnal hemoglobinuria (PNH) is an acquired clonal hemolytic anemia that is associated with other bone marrow failure syndromes including aplastic anemia and myelodysplastic syndrome [1]. Although PNH has recently been associated with *JAK2*^*V617F*^ positive myeloproliferative neoplasms [2] and benign mesenchymal tumors [3], it has not been associated with solid tumor malignancies, to our knowledge. We report an unusual case of a patient who presented with an occult primary tumor which was followed shortly thereafter by the development of profound hemolysis due to PNH.

## 2. Case Presentation

A 64-year-old Caucasian woman presented at an outside hospital in March 2009 with shortness of breath two days after undergoing laparoscopic cholecystectomy forcholedocholithiasis. A chest radiograph demonstrated bibasilar and left upper lobe pulmonary opacities. Computed tomography (CT) of the chest revealed mediastinal and right supraclavicular lymphadenopathy, with right basilar and upper lobe consolidation, but no evidence of pulmonary embolism. She was diagnosed with postoperative pneumonia and discharged with oral antibiotics.

**Figure 1:**
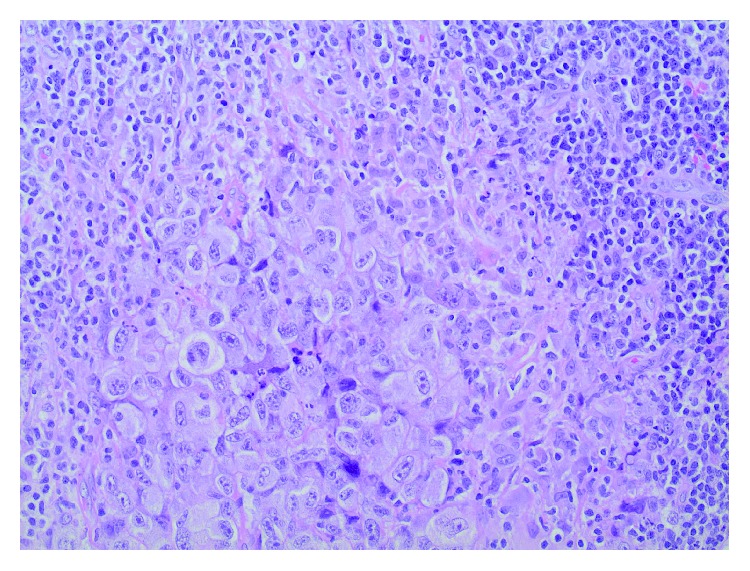
Lymph node biopsy. Sections show high-grade, pleomorphic malignant cells in a background of small lymphocytes (H&E, 200x).

**Figure 2:**
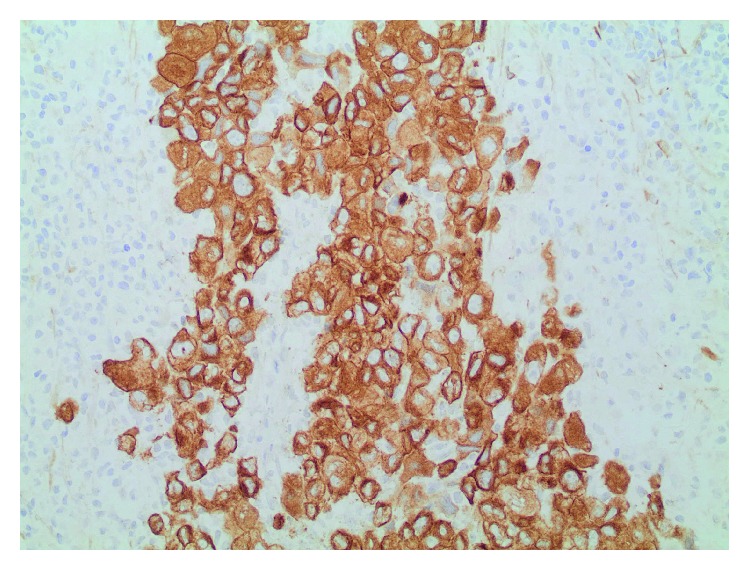
Lymph node biopsy. The malignant cells are strongly positive with antibodies to cytokeratin, indicating epithelial lineage and supporting the diagnosis of carcinoma (cytokeratin AE1/AE3, 200x).

A follow-up CT chest in July 2009 demonstrated the continuing presence of right supraclavicular lymphadenopathy, now measuring 3 cm, and additional 1.5 cm lymph nodes in the pretracheal and subcarinal regions. The patient underwent an excisional lymph node biopsy on July 24, 2009, that revealed replacement of the lymph node by highly pleomorphic cells with prominent nucleoli and abundant cytoplasm, suggestive of high-grade metastatic carcinoma (Figure 1). Immunohistochemical evaluation showed that 100% of the cells were positive for cytokeratin (CK) (Figure 2) but negative for CK7, CK20, thyroid transcription factor-1 (TTF-1), melanoma antigen (Melan-A), CD30, and leukocyte common antigen. A few cells were positive for S100. There was no positive nuclear staining reaction for the estrogen or progesterone receptors in any of the tumor cells. In the absence of morphologic or histochemical features to identify a primary site, a positron emission tomography (PET)/CT was performed. This revealed right lower cervical and right supraclavicular lymphadenopathy with intense hypermetabolism and moderate hypermetabolic activity in the right peritracheal and precarinal lymph nodes. Increased fluorodeoxyglucose (FDG) uptake in the region of the left lateral oropharyngeal wall and base of the tongue without definite CT correlation was also noted. In addition, heterogenous uptake in the marrow of the axial and proximal appendicular skeleton without correlation on bone windows was noted. Clinically, the patient denied any localizing or systemic symptoms of malignancy. She was a nonsmoker with minimal alcohol intake. Endoscopic evaluation of her upper airways and breast magnetic resonance imaging (MRI) were unrevealing for a primary site.

The patient was diagnosed with carcinoma of unknown primary and was prescribed 4 cycles of carboplatin and paclitaxel, which was completed in February 2010. She was then switched to maintenance erlotinib in May 2010. Six days after initiation of erlotinib, the patient presented with jaundice and passage of dark red urine. Laboratory studies revealed hemoglobin (Hgb) of 7.1 g/dL (range: 12.0–16.0 g/dL), platelet count of 92 × 10^3^/*μ*L (range: 150–450 × 10^3^/*μ*L), total bilirubin of 5.0 mg/dL (range: 0.2–1.0 mg/dL), and elevated liver enzymes and lactate dehydrogenase of 2,546 U/L (range: 81–234 U/L). The direct antiglobulin (Coomb's) test was negative. Urinalysis revealed a large amount of blood with zero RBC/hpf. The patient was started on prednisone 1 mg/kg for presumed erlotinib-induced hemolytic anemia. Bone marrow biopsy revealed a mildly hypercellular bone marrow with erythroid hyperplasia. The dose of erlotinib was reduced to 100 mg daily with waxing and waning of her hemolytic symptoms despite ongoing treatment with prednisone. Erlotinib was discontinued in July 2010, and the prednisone dose was tapered beginning in October 2010. In November 2010, the patient presented again with hemolytic anemia that failed to respond to high-dose prednisone. She was referred to our institution for further evaluation of her hemolysis.

She reported multiple episodes of intermittent coffee color urine in the preceding years, which resolved spontaneously. Urinalysis revealed 3+ hemosiderinuria. The highly sensitive flow cytometry assay (FLAER) revealed a PNH clone in 91% of granulocytes, 93% of monocytes, and 78% of red cells. With a confirmed diagnosis of PNH, prednisone was discontinued and eculizumab (900 mg every 2 weeks) was initiated. Treatment with eculizumab resulted in improvement of her laboratory values (Hgb 11.0 g/dL, white blood cells 4.6 × 10^3^/*μ*L with a normal differential, and platelets 135 × 10^3^/*μ*L) with cessation of hemoglobinuria.

In early 2011, the patient developed acute thrombocytopenia to <20 × 10^3^/*μ*L with no significant change in other cell lines. With other possible causes excluded, a diagnosis of immune thrombocytopenic purpura (ITP) was made. She was treated with rituximab 375 mg/m^2^ weekly for 4 weeks, with normalization of the platelet count. She has remained on eculizumab with no recurrence of hemolytic anemia.

With regards to the underlying malignancy, a staging CT in March 2011 showed minimal change in the previously noted right supraclavicular and mediastinal lymph nodes. Subsequent imaging in August 2011 demonstrated decreased prominence of the right supraclavicular and mediastinal lymphadenopathy with no other evidence of metastatic disease. As of April 2019, the patient remains on eculizumab 900 mg every 2 weeks, with no signs of cancer recurrence.

## 3. Discussion

Cancers of unknown primary (CUP), or occult primary tumors, are defined as histologically proven metastatic malignant tumors in which the primary site cannot be identified [4, 5]. These tumors confer a poor prognosis in most patients with a median survival of 8 to 12 months [6, 7]. As such, the fact that our patient with CUP remains disease-free more than 8 years after diagnosis is remarkable.

One possibility to explain this patient's long-term survival is the concomitant eculizumab for PNH management. Eculizumab is a humanized monoclonal antibody that binds to C5 and prevents its cleavage by C5 convertase, thereby inhibiting the generation of C5a and downstream formation of the membrane attack complex. In recent years, a substantial body of literature has linked complement factors C3a and C5a to various aspects of tumor biology. In addition to their recognized role in sustaining chronic inflammation, the anaphylatoxins C3a and C5a also promote a microenvironment that is immunosuppressive for tumor growth, is proangiogenic, and accelerates tumor growth through enhancement of tumor cell migration and subsequent tissue invasion and metastasis [8, 9]. Studies using animal models of lung cancer reported higher C5 deposition in lung cancer cell lines than in nonmalignant bronchial epithelial cells, thereby creating a favorable tumor microenvironment by inducing endothelial cell chemotaxis and blood vessel formation [10].

As such, the inhibition of C5 convertase by eculizumab could theoretically mediate an antitumor effect. Indeed, this has been demonstrated in several preclinical models of melanoma and lung cancer where pharmacologic inhibition of C5a potentiated blockade of the programmed cell death protein 1 (PD-1) pathway using immune checkpoint inhibitors [11, 12]. Animal models targeting complement pathways in cancer remain an area of active research. It would be of interest to determine if these findings could translate into clinical practice, given the favorable clinical outcomes seen using immunotherapy and checkpoint inhibitors in cancer therapy. In addition, given the traditional role of complement in enhancing the cytolytic effects of antibody-mediated immunotherapy, pharmacologic manipulation of complement will remain a challenge due to its uncertain benefit vs. harm in the cancer microenvironment [9].

In conclusion, our patient remains in prolonged remission for cancer of unknown primary following initiation of eculizumab therapy for PNH. Regardless of whether the co-occurrence of cancer and PNH was coincidental in this patient, the uniqueness of the case is emphasized by the remarkable and sustained response of not only the manifestations of PNH but also the underlying cancer, to eculizumab.
